# Contrasting patterns of sexually selected traits in Mediterranean and continental populations of European mouflon

**DOI:** 10.1002/ece3.6041

**Published:** 2020-01-24

**Authors:** Krešimir Kavčić, Luca Corlatti, Toni Safner, Nikola Budak, Nikica Šprem

**Affiliations:** ^1^ Department of Fisheries, Apiculture, Wildlife Management and Special Zoology Faculty of Agriculture University of Zagreb Zagreb Croatia; ^2^ Chair of Wildlife Ecology and Management University of Freiburg Freiburg Germany; ^3^ Department of Plant Breeding, Genetics and Biometrics Faculty of Agriculture University of Zagreb Zagreb Croatia; ^4^ Centre of Excellence for Biodiversity and Molecular Plant Breeding (CoE CroP‐BioDiv) Zagreb Croatia; ^5^ Zagreb Croatia

**Keywords:** environmental change, horn growth, reproduction, sexual selection, ungulates

## Abstract

The expression of sexually selected traits in highly dimorphic ungulates may be influenced by environmental quality. Variations in habitat conditions can impose different constraints on the allocation of energy resources to male life‐history traits, and possibly alter the female preferences for specific features. Here, we compared the horn growth patterns in male European mouflon *Ovis aries musimon* living in different habitats (Mediterranean vs. continental) but sharing a common genetic origin. We hypothesized that the expression of sexually selected traits such as horn development should be promoted in more favorable habitat conditions (i.e., Mediterranean). Using linear mixed models on data retrieved from individuals harvested under the same hunting regime, we found longer horns and greater individual variance in horn segment length in the Mediterranean population than in the continental one. Furthermore, Mediterranean rams showed no evidence of compensatory horn growth, as opposed to the continental rams. Unexpectedly, horn base circumference was greater in the continental habitat than in the Mediterranean one. The overall results suggest different patterns of investment in horns in the two populations, with seemingly stronger pressure and consequences of sexual selection on mouflon rams living in more favorable environments. Although the role of hunters' selectivity cannot be excluded a priori, our data suggest that the differences in the expression of sexually selected traits in our study populations may be influenced by environmental conditions. Because sexual selection can impose substantial fitness costs on individuals, further investigations on the trade‐offs between reproduction and survival would improve our understanding of the dynamics of mouflon populations living in different environmental conditions.

## Graphical abstract

The expression of sexually selected traits in highly dimorphic ungulates may be influenced by environmental quality. We compared the horn growth patterns in male European mouflon living in different habitats but sharing a common genetic origin. Our results suggest different patterns of investment in horns in two populations, suggesting that the differences in the expression of sexually selected traits may be influenced by environmental conditions.

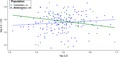

## 1 INTRODUCTION

Sexual selection is thought to be the main evolutionary driver of weaponry development in male ungulates (Andersson, 1994; Geist, 1966). In polygynous ungulates under strong pressure of sexual selection, horn size correlates positively with breeding success (e.g., red deer *Cervus elaphus*: Kruuk et al., 2002; bighorn sheep *Ovis canadensis*: Coltman, Festa‐Bianchet, Jorgenson, & Strobeck, 2002; alpine ibex *Capra ibex*: Willisch et al., 2012). Hence, males typically invest conspicuous amounts of energy in growing large weapons and tend not to compensate for shorter horn growth in early life, that is, early growth positively correlates to horn development later in life (Willisch, Biebach, Marreros, Ryser‐Degiorgis, & Neuhaus, 2015). In these species, individual heterogeneity in weapon growth may thus be important (Carvalho et al., 2017; Festa‐Bianchet, 2017; Toïgo, Gaillard, & Loison, 2013), leading to fast‐growing and slow‐growing individuals, with consequences on fitness components (Coltman et al., 2002; Robinson, Pilkington, Clutton‐Brock, Pemberton, & Kruuk, 2006). Conversely, in polygynous ungulates under relatively weaker pressure of sexual selection, male weapon size does not appear to be related to breeding success (e.g., mountain goat *Oreamnos americanus*: Mainguy, Côté, Festa‐Bianchet, & Coltman, 2009; Northern chamois, *Rupicapra rupicapra*: Corlatti, Lebl, Filli, & Ruf, 2012). In these species, individuals with relatively shorter horns in early life tend invest more in horn development in later years, that is, they show partial compensatory horn growth (Rughetti & Festa‐Bianchet, 2010). This mechanism of counter‐selection reduces individual heterogeneity in weapon growth, thus leading to horn length distributions with relatively shorter tails (Corlatti, Gugiatti, & Imperio, 2015).

In bovids, horns grow continuously throughout an animal's life and, besides the evolutionary pressure exerted by competition over mating, their development can be modulated by a number of other factors, either external or internal. Horn growth and size, for example, can be influenced by climate (von Hardenberg, Bassano, del Pilar Zumel Arranz, & Bogliani, G., 2004), geological substrate and topography (Chirichella, Ciuti, Grignolio, Rocca, & Apollonio, 2013), or by vegetation communities (Festa‐Bianchet, Coltman, Turelli, & Jorgenson, 2004). Other external selective pressures include trophy hunting (Douhard, Festa‐Bianchet, Pelletier, Gaillard, & Bonenfant, 2016; Festa‐Bianchet, Pelletier, Jorgenson, Feder, & Hubbs, 2014; Garel et al., 2007) and population density (Douhard et al., 2017; Jorgenson, Festa‐Bianchet, & Wishart, 1998; Kavčić, Corlatti, Safner, Gligora, & Šprem, 2019). Internal factors such as hormones secretion (Santiago‐Moreno et al., 2005; Toledano‐Díaz, Santiago‐Moreno, Gómez‐Brunet, Pulido‐Pastor, & López‐Sebastián, 2007) and genetic variability (Geist, 1971; von Hardenberg et al., 2007) are also considered key drivers of horn development. In addition, recent research suggests that horn growth may be positively affected by hybridization events (i.e., in Northern chamois: Kavčić et al., 2018).

In male bovids under strong pressure of sexual selection (e.g., bighorn sheep: Coltman et al., 2002), weaponry can be energetically demanding to produce and maintain. Intuitively, the expression of this sexual trait, either in terms of absolute size, compensatory growth or individual heterogeneity, can be considered condition‐dependent, that is, it can be highly variable in populations living in different environmental conditions (cf. Rowe & Houle, 1996; Zahavi, 1975). Specifically, sexual selection is expected to be weaker under mild or harsh conditions and stronger under moderate levels of stress, which may enhance expression of genetic variation in individual condition (Hoffmann & Merila, 1999). Recent works pointed out that variation in environmental quality is important in determining the strength of sexual selection, whose effects may alter the rate of adaptation to new environments, indirectly affecting population dynamics and viability (Candolin & Heuschele, 2008; Martínez‐Ruiz & Knell, 2017). For example, qualitative environmental changes can disrupt the preference for a specific sexual trait by reducing its benefit when the link between trait value and individual quality is altered (Wong, Candolin, Lindström, 2007). In addition, the adjustment of trait expression to new environmental conditions is usually followed by phenotypic plasticity, which may change cost and benefits of sexually selected traits (Kokko & Heubel, 2008a; Kokko & Heubel, 2008b). The strength of sexual selection should thus not be considered as a fixed, species‐specific characteristic, as it may vary depending on local environmental conditions (Martínez‐Ruiz & Knell, 2017).

The European mouflon *Ovis aries musimon* is a highly dimorphic caprid that originated in western Asia (Groves & Grubb, 2011), in areas with hot and dry summers and mild winters, but it has been introduced to many European countries (Bon et al., 1991). Despite its adaptability to different environments, the European mouflon maintains a preference for typically Mediterranean shrubby and rocky areas (Pfeffer, 1967), where it feeds on a high diversity of plants, including many species of shrubs (Marchand et al., 2013). Notwithstanding its importance for consumptive purposes, little is known about the horn growth pattern in European mouflon and the condition‐dependent investment in secondary sexual traits (but see: Garel et al., 2007; Lincoln, 1998). Here, we aim to compare the male horn growth patterns of two hunted populations introduced to Mediterranean and continental parts of Croatia. These two populations live in a very different environmental condition, but share the same founders and the same hunting regime.

Because European mouflons are highly polygynous, thus under strong pressure of sexual selection, we expect the expression of sexually selected traits to differ in males living in different habitats. Specifically, we hypothesized that the opportunity to invest in weapon growth should be greater in more favorable and stable environments, such as the Mediterranean one, compared with less favorable and more stochastic environments, such as the continental one (cf. Kokko & Brooks, 2003). Greater food availability should increase the strength of sexual selection (Janicke, David, & Chapuis, 2015), thus positively affect phenotypic and genetic variance of condition‐dependent sexually selected traits (David, Bjorksten, Fowler, & Pomiankowski, 2000). Specifically, we predicted that:

1. Male horn length and base circumference size should be greater in the Mediterranean than in the continental population (i.e., stronger sexual selection should favor larger horns).
2. There should not be evidence for compensatory horn growth in males in the Mediterranean population, because relative resource allocation to a specific trait should increase under good environmental and nutritional conditions (Jobling, 2010). In contrast, compensatory growth mechanism might activate when conditions are less favorable (i.e., in the continental area), thus reducing resource allocation to a specific trait (i.e., greater sexual selection should promote a positive relationship between horn annuli in the most favorable area, and partly negative relationship in the less favorable area).
3. Individual heterogeneity in male horn growth should be greater in the Mediterranean than in the continental population (i.e., stronger sexual selection should favor differences among individuals in the most favorable area).

## 2 MATERIALS AND METHODS

### 2.1 Study areas and populations

**Figure 1:**
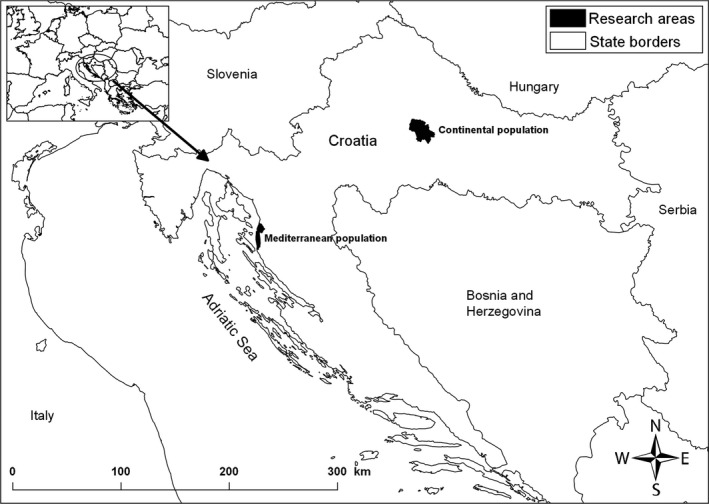
Location of the study populations of European mouflon in western (Mediterranean) and central (continental) Croatia

The study was conducted in two areas of Croatia, hosting two separate populations of European mouflon. The northern Dinaric mountains, hereafter “Mediterranean population”, and central Croatia, hereafter “continental population” (Figure 1). These populations show major differences in environmental and climatic features (Table 1), with milder and more stable (i.e., more favorable, for European mouflon) environmental conditions in the Mediterranean habitat than in the continental habitat (Seletković, Tikvić, Vučetić, & Ugarković, 2011). Both populations were introduced in the 1980s: Notably, the same source population from Brijuni islands was used for introduction (Kusak & Krapinec, 2010), thus the study populations share the same genetic origin. Today, both populations appear to be numerically stable with similar population sizes, at ca. 300 individuals (Anonymous, 2019a). Population size was estimated by visual counts, performed by hunter during field observations twice a year (in spring and in autumn).

**Table 1:** Main environmental and climatic characteristics (mean values) of the study areas inhabited by the Mediterranean and continental European mouflon populations

	Population	
	Mediterranean	Continental
Elevation (m. a.s.l.)	226	200
Substrate	Calcareous	Calcareous
Spring temp. (°C)	19.2	14.7
Summer temp. (°C)	24.6	18.8
Winter temp. (°C)	7.2	2.2
Open areas (%)	85	25
Vegetation	Low form (macchia)	High form (broadleaf forests)
Sun hours	2,338	1,740
Days with snow ≥1cm	<5	30–50
Spring precipitation (mm)	256	85

In both areas, several ungulate species coexist with European mouflon: roe deer *Capreolus capreolus*, fallow deer *Dama dama,* and wild boar *Sus scrofa* (Krapinec, Mičija, Bukovinski, & Pintur, 2013). Chamois can be found only in the Mediterranean area and red deer in the continental one.

### 2.2 Data collection

Horn growth data were recorded on 514 samples (*n* = 431 for the Mediterranean and *n* = 83 for the continental population) of European mouflon rams legally culled based on game management plans between 1985 and 2012 in the Mediterranean population, and between 1992 and 2002 in the continental population. Age was determined by counting of horn annuli (Geist, 1966), whereas horn measurements were taken on both horns. The absence of directional asymmetry in Caprinae species is evident (von Hardenberg et al., 2004) and since most horns had some wear on the tip, we considered only the longest horn to avoid errors in data analysis. We used a flexible ruler to measure base circumference and each annulus from the first (L1) to the tenth (L10), depending on the animal's age. Only complete segments were considered for analysis. Measurements were taken only once by the same experienced game warden using a standardized methodology (Merchant, Hoefs, Nette, Kale, & Janssen, 1982). The hunting regime is the same in both areas, as individuals are hunted in accordance with pre‐defined game management plans (J. Tomljanović, personal communication), and regulated by the Croatian hunting act (Anonymous, 2019b).

### 2.3 Data analysis

We created two datasets to be used in the analyses. The first (full) dataset consisted of the horn length measures for all 514 individuals, used for the analysis of total horn length and base circumference, and to assess compensatory growth. The second (reduced) dataset included only individuals from cohorts present in both populations, that is, cohorts (year of birth) between 1987 and 1999 (*n* = 183 for the Mediterranean and *n* = 83 for the continental population) to test for individual heterogeneity within the same cohorts.

To test for the influence of the environmental quality, hereafter referred to as “habitat” (Mediterranean vs. continental), on the total horn length and base circumference (response variables), we fitted separate linear mixed models (LMMs) for both response variables. The predictors included in both models were habitat (2‐levels fixed nominal variable), individual age at death and cohort (factors with random intercepts fitted for each age and cohort classes).

To explore the potentially different patterns of compensatory growth between the two populations, we first selected from the full dataset individuals that had at least 5 years of age at the time of harvest (*n* = 169 for the Mediterranean and *n* = 44 for the continental population). We then fitted a linear model to regress the cumulative length of horn segments L3–L5 (response variable) against the length of the second horn segment (L2) corrected for cohort and age, with population fitted as categorical predictor. Since all models were conceptually developed prior to analyses, following the methodological framework used in previous studies (Alpine ibex: Toïgo et al., 2013; chamois: Corlatti et al., 2015; Kavčić et al., 2018), we did not use any model selection procedure.

Lastly, to investigate the importance of individual heterogeneity on log‐transformed horn segment length variation (response variable), for each population, we fitted a linear model with cohort and age of the segment as fixed variables, while animal identity was fitted as a factor with random intercepts for each individual. Fixed factors accounted for the variation of horn segment lengths between different cohorts and variation between horn segment lengths between segments grown in different animal ages. The individual random factor was included to assess the correlation among repeated measures of horn annuli within the same individual, that is, the intraclass correlation coefficient (ICC). ICC provides information on the degree of similarity of the annuli length within individuals, relative to the difference in annuli length among individuals. In simpler words, ICC allows to measure repeatability that is the level of individual clustering in horn annuli length: the higher the ICC value, the greater the clustering (i.e., the higher the difference among individuals in terms of horn annuli), thus the more variance is explained by individual heterogeneity. To estimate this variance, we calculated the conditional and marginal *R* ^2^ statistics developed by Nakagawa and Schielzeth (2013) for each population model. The difference between the two statistics represents the variance explained by the individual random factor within each population. In addition, for each area we estimated the “adjusted” repeatability, that is, the repeatability (ICC) values obtained from the fitted models after accounting for fixed effects (Stoffel, Nakagawa, & Schielzeth, 2017).

Statistical significance was set at *p* ≤ .05 for all tests. All statistical analyses were performed in R 3.3.2 (R Core Team, 2016) in RStudio 1.1.423 (RStudio Team, 2016), using the “lme4” package (Bates, Mächler, Bolker, & Walker, 2015) for LMM and the “MulMln” package (Barton, 2013) for modeling and estimation of *R* ^2^ statistics. Repeatability of models was estimated using the function rpt in the “rptR” package (Stoffel et al., 2017).

## 3 RESULTS

**Figure 2:**
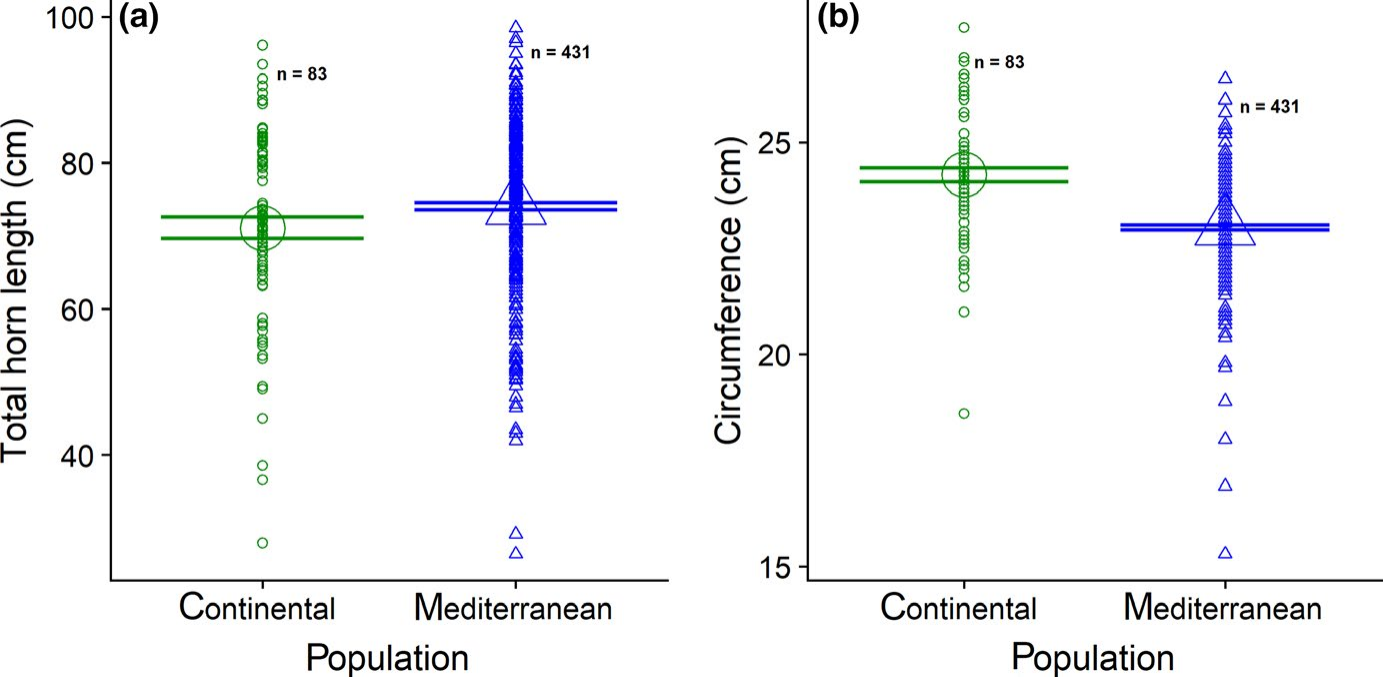
(a) Total horn length (in cm) and (b) basal horn circumference (in cm) in the Mediterranean and continental European mouflon populations between 1985 and 2012. Values are means and standard deviations

The effect of population, corrected for age and cohort, was significant for total horn length (*F* = 134.28; *p* < .001) and base circumference (*F* = 31.84; *p* < .001). Specifically, horns were longer in the Mediterranean habitat (Mediterranean: least square mean = 80.16, *SE* = 3.80; continental: least square mean = 70.66, *SE* = 3.84) whereas circumferences were slightly bigger in the continental habitat (continental: least square mean = 23.86, *SE* = 0.25; Mediterranean: least square mean = 23.00, *SE* = 0.25). This suggests different patterns of investment in horns in different populations (Figure 2).

**Figure 3:**
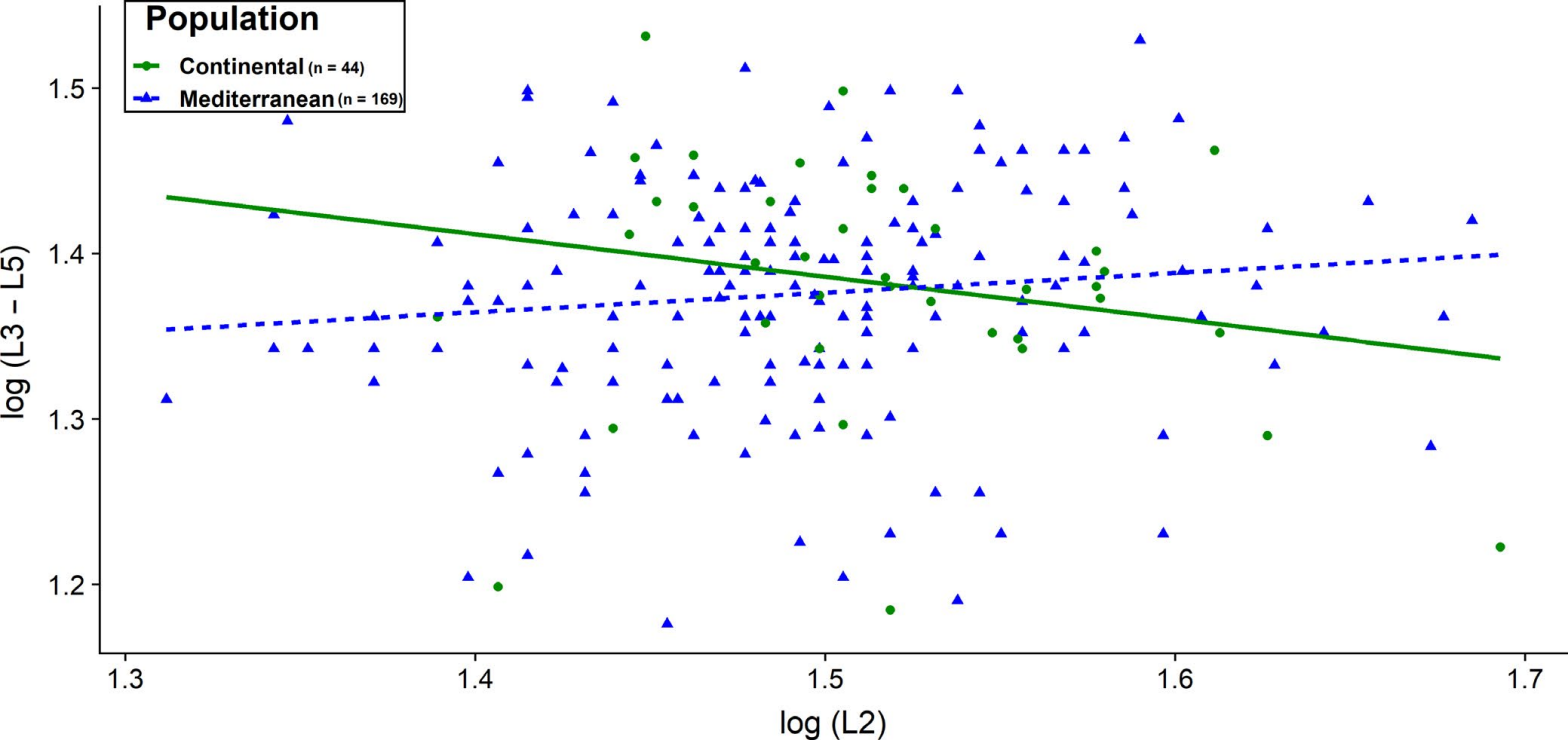
Relationship between log‐transformed second horn segment length L2 and log‐transformed cumulative length of horn segments L3–L5 in the Mediterranean and continental European mouflon populations between 1985 and 2012

We did not find a significant effect of population and early horn growth (L2) on the sum of segment length from L3 to L5 (*p* > .05, Figure 3), thus no significant compensatory growth in either population. Yet, we found a weak signal for partial horn growth compensation in the continental area, suggesting that rams in this population may have invested less energy in growing large horns than did the Mediterranean ones.

The effect of individual heterogeneity on segment length was highly significant in the Mediterranean population (individual ID: LRT = 27.706, *p < *.001), but not in the continental population (individual ID: LRT = 0.034, *p > *.05). The proportion of variance in horn segment length explained by individual heterogeneity differed between populations (Mediterranean: marginal *R* ^2^ = .86, conditional *R* ^2^ = .89; continental: marginal *R* ^2^ = .80, conditional *R* ^2^ = .81), suggesting that individual horn growth was more diverse in the Mediterranean than in the continental population. Accordingly, the value of individual repeatability was higher in the Mediterranean population (estimate = 0.205, 95% CI: 0.126, 0.301), than in continental population (estimate = 0.068, 95% CI: 0.00, 0.241), thereby confirming that individual horn growth was more diverse in the former than in the latter.

## 4 DISCUSSION

Our data suggest that habitat characteristics may affect the patterns of sexually selected traits in European mouflon. A potential for environment‐dependent pressure of sexual selection may be indicated by different investments in horn growth between populations. Mediterranean rams had longer horns, showed no compensatory horn growth, and had greater individual heterogeneity in horn segment length compared with the continental ones. As the strength of artificial selection due to hunting pressure appears similar in both areas, our results suggest that the effects of sexual selection may be stronger in more favorable environments, thereby supporting our predictions.

Despite the shared genetic origin, mouflon rams in the Mediterranean population apparently invested more energy in weapon length (i.e., horn length and annual segments) than rams in the Continental population, which seemingly traded shorter horn lengths for slightly larger base circumferences. These data are in agreement with Krapinec et al. (2013), who found thicker base circumferences in the continental population and longer horns in the Mediterranean population. These authors suggested that different forage availability was the most important factors shaping horn growth between two study sites. Indeed, lower quality and availability of food resources are known to cause decline in horn size in European mouflon (Garel et al., 2007). This may result from a trade‐off between investment in survival (natural selection) and reproduction (sexual selection). In fact, allocation of energy to secondary sexual traits in the Caprini tribe is under strong influence of habitat conditions and varies with resource availability (Faliu, Cugnasse, Auvray, Orliac, & Rech, 1990; Festa‐Bianchet et al., 2004; Garel et al., 2007; von Hardenberg et al., 2004). The greater horn length found in the Mediterranean rams may thus support prediction (a) of greater pressure of sexual selection in this population than in the continental one, owing to relatively better environmental conditions. It remains unclear why continental rams invested more in horn circumference than the Mediterranean rams, especially because male horn base typically correlates with male horn length in wild sheep (Pigeon, Festa‐Bianchet, Coltman, & Pelletier, 2016).

The compensatory rates did not differ significantly between the two populations. Our data therefore support previous studies on wild sheep species, where compensatory mechanism was weak or absent (Festa‐Bianchet, 2017). Interestingly, however, we found a weak signal of compensation in horn growth in the continental population. This tendency might support prediction (b) that in relatively harsher environmental conditions, European mouflon shows a trade‐off in the investment between survival and reproduction. This would imply different effects of sexual selection in the two populations. Allocation of energy to horn development may be too costly for the continental rams when they have to face harsh environmental conditions. Consequently, individuals may compensate by redirecting their energy reserves to other important physiological processes and traits, such as body mass (Mason, Apollonio, Chirichella, Willis, & Stephens, 2014). In turn, this suggests a more conservative strategy of energy allocation in the continental population. In contrast, in the Mediterranean population some rams may afford to invest conspicuously in early horn growth, avoid compensatory growth, and because sexual selection normally favors large male size through rapid early growth (Andersson, 1994), they may possibly gain a reproductive advantage over smaller‐horned males (Coltman et al., 2002).

As a consequence of seemingly different patterns of compensatory growth, we found greater individual heterogeneity in horn segment length was in the Mediterranean population than in the continental one. This suggests higher frequency of “extreme” phenotypes (e.g., “low‐quality” and “high‐quality” individuals) in the former than in the latter population. In line with prediction iii), given that the investment in horn growth is energetically demanding (Zahavi, 1975), the opportunity to express extreme sexually selected traits is expected to increase in habitats of higher quality. The occurrence of great individual variability in horn length in both populations is not unexpected, as the effect of individual heterogeneity in Caprine species may explain more than 20% of the total variability in horn length (cf. Bergeron, Festa‐Bianchet, Hardenberg, & Bassano, 2008; Carvalho et al., 2017).

The different horn growth patterns observed in our study sites suggest that the expression of sexually selected traits in European mouflon may be modulated by environmental conditions. The potential role of trophy hunting in explaining our results, however, should not be discarded. For example, it is reasonable to assume that horn length—rather than horn base—is a direct target of trophy hunting (cf. Pigeon et al., 2016). If so, and if continental hunters were less selective than the Mediterranean ones, different hunting selectivity might create a sampling bias (i.e., longer‐horned rams would be shot preferentially in the Mediterranean population) which, however, is difficult to quantify (Mysterud, Trjanowski, & Panek, 2006), and thus contribute to explain our results. Even if the hunting regime adopted in two study areas was legally the same, we cannot exclude the possibility that the hunters might have had slightly different preferences toward larger or smaller rams. Nonetheless, preliminary data suggest that no significant difference in selectivity (i.e., age at culling vs. early horn growth, cf. Douhard et al., 2016) exists between the two populations. Furthermore, our suggestion that habitat quality may be the major driver of the observed differences is supported by previous studies showing that forage supply had a positive impact on horn volume, but only in the continental population (Krapinec et al., 2013). Additionally, the effect of founders’ morphological traits might also play a role in shaping contrasting horn growth patterns in the study populations; no data are available to clarify whether this stochastic effect might have had an impact on our results.

Sexual selection can impose substantial fitness costs at the individual level, especially in low‐quality habitats (Janicke et al., 2015; Martinossi‐Allibert, Rueffler, Arnqvist, & Berger, 2019), with consequences for the dynamics of populations. We acknowledge that our study does not explicitly investigate the role of potential environmental drivers on the pattern of sexually selected traits. Future research should thus aim to elucidate the fine‐scale mechanisms linking sexual traits and environmental constraints, thus the trade‐offs between reproduction and survival in mouflon living in different environmental conditions. This, in turn, should allow to better understand the consequences of such trade‐offs for the dynamics and thus the management of populations.

## CONFLICT OF INTERESTS

We have no competing interests.

## AUTHORS' CONTRIBUTION

KK wrote all drafts of the manuscript. LC conceptualized the framework and revised all drafts of the manuscript. TS did the statistical analyses. NB obtained and arranged raw data. NS supervised all stages of this work, from data collection to data analysis, and participated in revising the manuscript.

## Data Availability

Data used in this analysis are available at Dryad Digital Repository: https://doi.org/10.5061/dryad.n5tb2rbrx.
